# Quality and Reliability of YouTube Videos on Poisonings, Insect Bites, and Envenomations

**DOI:** 10.3390/healthcare13243224

**Published:** 2025-12-10

**Authors:** Ali Halici, Behçet Demir, Çağla Özdemir

**Affiliations:** 1 Medical School, Kutahya Health Sciences University, Kutahya 43300, Turkey; 2 Kutahya Evliya Celebi Training and Research Hospital, Kutahya 43040, Turkey

**Keywords:** YouTube videos, poisoning, web-based health information

## Abstract

**Background**: YouTube has become one of the most widely used platforms for medical education and patient information. However, the accuracy and reliability of such unregulated content remain highly variable and sometimes misleading. This study aimed to evaluate the quality, reliability, and educational value of YouTube videos related to poisonings, insect bites, and envenomations using validated scoring systems. **Methods**: A cross-sectional analysis of YouTube videos was conducted using the search terms “approach to insect bites and stings,” “approach to poisonings,” “approach to scorpion envenomation,” “approach to snake envenomation,” and “approach to mushroom poisoning.” Searches were performed in incognito mode on August 1, 2025. Only English-language videos shorter than one hour were included. Video quality and reliability were evaluated using the Global Quality Score (GQS), modified DISCERN (mDISCERN), and Journal of the American Medical Association (JAMA) benchmarks, while viewer engagement was measured using the Video Power Index (VPI). **Results**: A total of 279 videos were analyzed. The mean ± SD scores were as follows: GQS, 3.53 ± 1.09; mDISCERN, 3.53 ± 1.08; and JAMA, 2.63 ± 0.96. Based on the GQS, 59.5% of the videos were high quality, 20.8% moderate quality, and 19.7% low quality; thus, approximately 40% of the evaluated videos (low- and moderate-quality categories combined) did not meet optimal quality standards. Videos on snake envenomation and general poisoning had significantly higher quality and reliability scores (*p* < 0.001). Educational, physician-sourced, and physician-presented videos achieved higher GQS, JAMA, and mDISCERN values (*p* < 0.001 for all). However, no significant differences were found in the VPI, indicating that popularity metrics did not correlate with content quality. **Conclusions**: YouTube provides wide access to poisoning-related educational materials, but content quality varies considerably, and a substantial proportion of videos fall below acceptable quality thresholds. Videos produced or presented by physicians are more reliable, whereas popularity is not a valid indicator of scientific accuracy. Active involvement of healthcare professionals and academic institutions, together with platform-level quality verification and visibility strategies, is essential to improve the credibility and impact of online health information.

## 1. Introduction

In the modern era, the abundance of information sources can often be more confusing than the lack of information itself. Internet users are now required to evaluate the reliability of widely accessible health content [1]. The Internet has become a major source of medical knowledge; however, 82% of patients rarely or never discuss the information they find online with their physicians. YouTube, a free and non-peer-reviewed video-sharing platform, accounts for approximately 60% of all online video views, making it the third most visited website worldwide [2].

Previous studies evaluating the quality of medical content on YouTube have primarily focused on cardiovascular emergencies, dermatologic conditions, or general health topics. However, the quality of videos addressing toxicology and poisoning conditions where timely and accurate information is essential remains largely understudied. Existing research also tends to provide descriptive summaries rather than critical comparisons of validated scoring systems. Moreover, very few studies have evaluated poisoning-related videos using multiple established tools such as GQS, JAMA, and mDISCERN simultaneously.

Recent studies have highlighted substantial variability in the quality of online health information and the growing influence of digital platforms on public health behaviors. For example, Anastasio et al. reported poor quality among TikTok videos related to ankle sprain rehabilitation [3], while Xiao et al. demonstrated that video-based health promotion can be effective but varies widely in methodological rigor [4]. In addition, broader social media research shows significant implications for public health and mental well-being, particularly among young users [5]. These studies collectively underscore the need for systematic evaluation of online medical content. However, poisoning-related videos where misinformation may have immediate clinical consequences remain critically understudied. Our work seeks to address this gap.

Social media platforms, particularly YouTube, have become accessible tools for both professional medical education and patient experiences [6,7]. YouTube hosts numerous high-quality and useful educational materials [8]. Mamlin et al. predicted that such platforms would play an important role in health information exchange and public health communication [9]. Indeed, 88% of users report searching for health information online, and nearly half make medical consultation decisions based on this content [10]. However, the general public may lack the ability to distinguish reliable medical videos from misleading ones, emphasizing the need for evaluating online content quality [8].

Poisoning represents a major public health concern, ranking among the leading causes of injury-related deaths in the United States, with more than 40,000 deaths annually. About 2.4% of emergency department injury visits are due to poisoning. Despite the availability of poison control centers and toxicology consultation, emergency physicians are often responsible for the initial evaluation. Insufficient risk assessment or lack of basic toxicology knowledge may jeopardize patient safety [11].

YouTube attracts vast public attention, with over 500 h of video uploaded every minute and one billion hours of viewing daily [12,13,14]. Although this provides broad access to health information, inconsistencies, lack of peer review, and complex terminology remain major drawbacks [15]. Since anyone can upload content without verification, the risk of misinformation increases significantly [16].

To address these concerns, several validated scoring systems such as the modified DISCERN (mDISCERN), Journal of the American Medical Association (JAMA) criteria, and Global Quality Score (GQS) have been developed to assess the reliability and educational value of online health videos [17,18,19].

This study aimed to evaluate the content quality and reliability of YouTube videos related to poisonings, insect bites, and snake or scorpion envenomations, and to classify the sources of these videos. Additionally, by comparing quality scores across educational, source, and speaker categories, this study sought to highlight the impact of professional content on informational accuracy.

## 2. Materials and Methods

### 2.1. Ethics Statement

This study analyzed publicly available videos on the international social networking platform YouTube. Since no human or animal participants were involved and all materials were publicly accessible, institutional ethics committee approval and patient consent were not required.

### 2.2. Video Search on YouTube

The official YouTube website (https://www.youtube.com) was used as the data source. Searches were performed on 1 August 2025 using the terms “approach to insect bites and stings,” “approach to poisonings,” “approach to scorpion envenomation,” “approach to snake envenomation,” and “approach to mushroom poisoning.” The selection of these keywords was guided by two factors: first, they reflect standard terminology commonly used in emergency medicine and toxicology guidelines, and second, a preliminary keyword exploration using YouTube’s autocomplete function indicated that these phrases are frequently searched by general viewers seeking poisoning-related information. To minimize algorithmic bias, all searches were conducted in incognito mode without signing into a Google account, and videos were sorted using YouTube’s default relevance algorithm, which incorporates factors such as views, likes, recency, and user engagement. Only English-language videos shorter than one hour were included in the analysis, while non-English and longer videos were excluded.

**Figure 1:**
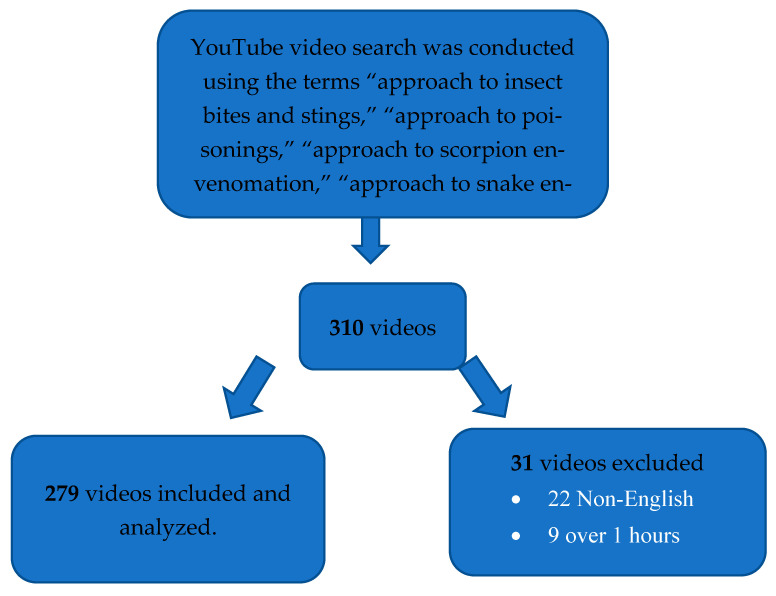
Flow Diagram of the YouTube Video Selection Process.

A total of 310 videos were screened. Twenty-two non-English videos and nine videos longer than one hour were excluded, leaving 279 eligible English-language videos for analysis (Figure 1).

### 2.3. Video Metadata

Each video was analyzed for several parameters, including number of views, upload date, daily view count, duration, source, number of likes, and number of comments.

### 2.4. Video Sources

Video sources were categorized into seven groups: (1) physicians or physician groups, (2) universities/professional associations/non-profit organizations, (3) governmental or news agencies, (4) independent health information websites, (5) medical advertisements or for-profit companies, (6) individuals, and (7) others.

### 2.5. Video Quality Analysis

Two independent emergency medicine specialists working in a tertiary-care emergency department evaluated all videos, and the mean of their scores was used for analysis. Each video was assessed in terms of poisoning-related definitions, complications, clinical signs/symptoms, and treatment content.

In line with recent social media engagement studies [20], we included multiple interaction-based metrics such as number of likes, comments, total views, and view ratio (views/day). These variables were analyzed alongside quality scores to explore whether user engagement aligns with scientific accuracy.

Previous studies have utilized various quality rating tools (QRTs) for video evaluation, categorized as externally validated, internally validated, or limited-global instruments. Among these, the Journal of the American Medical Association (JAMA) score, Global Quality Score (GQS), and modified DISCERN (mDISCERN) are externally validated and widely accepted tools. Accordingly, GQS and JAMA were used to assess video quality, while mDISCERN was employed to evaluate reliability.

Global Quality Score (GQS): GQS is a five-point Likert scale evaluating information quality, flow, and usefulness to patients. Scores range from 1 (poor quality) to 5 (excellent quality). A higher score indicates better educational value and overall quality.

JAMA Score: The JAMA benchmark assesses four criteria: authorship (affiliation, credentials), attribution (references, copyright), disclosure (ownership, sponsorship, advertisement), and currency (publication or update date). Each criterion is scored as 1 point, with a total possible score of 4.

Modified DISCERN (mDISCERN): Originally developed by Charnock et al. [21] this five-item tool evaluates the reliability of health information. Each “yes” response scores one point, with a total score range of 0–5. The questions assess the following:

1. The video is clear and understandable.
2. Valid sources are cited.
3. The information is balanced and unbiased.
4. Additional information sources are provided.
5. Controversial or uncertain areas are discussed.

Video Popularity Index (VPI): Video popularity was quantified using the Video Power Index (VPI), calculated as follows:

VPI = Like ratio × View ratio (daily views/100)

### 2.6. Statistical Analysis

All analyses were performed using IBM SPSS Statistics version 21 (IBM^®^, Chicago, IL, USA). The Shapiro–Wilk test was applied to assess the normality of data distribution. Descriptive statistics were expressed as the mean ± standard deviation or median (minimum–maximum), as appropriate. The Mann–Whitney U and Kruskal–Wallis tests were used for comparisons of non-normally distributed variables. Nominal data were compared using the chi-square test. Correlations between non-normally distributed variables were examined using Spearman’s correlation analysis. A *p*-value < 0.05 was considered statistically significant.

All videos were independently assessed by two emergency medicine specialists. Any discrepancies between reviewers were resolved by consensus after joint re-evaluation. Inter rater reliability was calculated using Cohen’s kappa statistic, demonstrating substantial agreement across all scoring systems. The kappa values were 0.87 for mDISCERN, 0.84 for JAMA, and 0.89 for GQS, indicating high consistency between evaluators.

## 3. Results

After excluding 31 videos (22 non-English and 9 longer than one hour), a total of 279 videos were included in the final analysis. The general characteristics and quality scores of these videos are summarized in Table 1.

Among all videos analyzed, 18.3% were related to scorpion envenomation, 22.9% to insect bites, 19.4% to mushroom poisoning, 19.0% to snake envenomation, and 20.4% to general poisoning approaches. The mean (±SD) number of views was 610,701 ± 4,086,360, and the mean time since upload was 1838 ± 1237 days. The mean daily view ratio was 413 ± 3428, with an average video duration of 10.53 ± 12.06 min, 4321 ± 25,268 likes, and 420 ± 4772 comments.

The mean (±SD) GQS, mDISCERN, and JAMA scores were 3.53 ± 1.09, 3.53 ± 1.08, and 2.63 ± 0.96, respectively. Based on GQS classification, videos were divided into three quality groups: low (1–2 points), moderate (3 points), and high (4–5 points). Accordingly, 19.7% of videos were of low quality, 20.8% of moderate quality, and 59.5% of high quality (Table 1).

When analyzed by category, the majority of videos (76%) were educational, followed by news/politics (12.2%), entertainment (7.2%), and interviews/blogs (4.3%) (Table 2). Based on video sources, physicians or physician groups accounted for the largest share (42.3%), followed by independent health information websites (14%), government/news agencies (13.3%), and universities or professional organizations (10.8%).

Regarding the speakers, 53.8% of the videos featured physicians, 20.4% reporters, 11.1% social influencers, 9% nurses, 1.8% caregivers or family members, and 1.4% patients (Table 2).

Table 3 presents the comparison of mean quality scores (GQS, JAMA, mDISCERN) and engagement metrics (VPI) among YouTube videos addressing different poisoning categories. Videos on snake envenomation and general poisoning demonstrated significantly higher GQS, JAMA, and mDISCERN scores compared to other groups (*p* < 0.001), indicating superior reliability and educational value. Although the VPI values varied among groups, the differences did not reach statistical significance (*p* = 0.060), suggesting that popularity metrics were not directly associated with content quality.

As shown in Table 4, the content quality and engagement levels of YouTube videos were compared across educational vs. non-educational, physician vs. non-physician sources, and physician vs. non-physician speakers. Video quality was evaluated using the modified DISCERN (mDISCERN), Journal of the American Medical Association (JAMA), and Global Quality Score (GQS) criteria, while viewer engagement was assessed with the Video Power Index (VPI).

Videos classified as educational, uploaded by physician sources, or presented by physician speakers demonstrated significantly higher mDISCERN, JAMA, and GQS values in all comparisons (*p* < 0.001 for each). However, no significant difference in VPI was found among these groups, suggesting that content popularity did not necessarily correlate with educational quality.

## 4. Discussion

YouTube has become an increasingly prominent source of health information; however, the quality and reliability of its medical content remain highly inconsistent and variable. This study systematically assessed the quality and reliability of YouTube videos related to poisoning, insect bites, scorpion and snake envenomation, and mushroom poisoning using validated tools, including the Global Quality Score (GQS), modified DISCERN, and JAMA benchmarks. The findings revealed marked heterogeneity across topics and sources: while approximately 60% of videos were of high quality, nearly 40% fell within the moderate- or low-quality range. Videos concerning snake envenomation and general poisoning scored significantly higher, suggesting uneven dissemination of health-related educational content.

The mean GQS (3.53 ± 1.09), mDISCERN (3.53 ± 1.08), and JAMA (2.63 ± 0.96) values align with previous studies reporting moderate educational value and reliability of medical videos. Krakowiak et al. found that most carbon monoxide poisoning videos lacked comprehensive or updated treatment information [22]. Similarly, Mukhamediyarov et al. reported that 25–30% of health-related videos contained misleading information, consistent with our observation that popularity indicators (VPI) do not necessarily reflect accuracy [23]. Misleading videos often gained similar or even greater engagement than high-quality ones. Sahin and Seyyar also observed low-to-moderate quality in chemotherapy videos, particularly those produced by commercial entities [7]. Similar results have been shown in pediatrics, where physician- or organization-generated videos performed better than those from independent users [24]. These patterns reinforce not only the importance of healthcare professionals in producing credible content, but also the necessity of institutional oversight in digital health communication.

Differences among poisoning categories were notable. Videos on snake envenomation and general poisoning achieved the highest quality scores, possibly reflecting greater global attention, availability of standardized treatment guidelines, and higher clinical relevance. In contrast, topics such as scorpion stings and mushroom poisoning received less engagement and often lacked detailed medical information. This pattern parallels findings in other rare or underrepresented medical conditions, such as those reported by Bolac et al. regarding Fuchs Endothelial Corneal Dystrophy [25].

VPI analysis indicated that while some high-quality videos achieved higher engagement (e.g., snake envenomation), overall popularity metrics (views, likes, comments) were not consistently aligned with educational quality. This aligns with the conclusions of Mukhamediyarov et al. that algorithm-driven popularity may amplify misleading content [23]. Similarly, Mohamed and Shoufan highlighted the misconception that view counts or likes imply reliability [10].

Consistent with earlier research [26,27], this study confirmed that educational, physician-sourced, and physician-presented videos scored significantly higher in mDISCERN, JAMA, and GQS, whereas the VPI showed no significant difference. Low-quality health content, however, often achieved higher visibility [28,29]. These findings underscore the need to systematically evaluate online medical content and promote the dissemination of evidence-based, peer-reviewed materials.

The findings highlight critical implications for clinical practice and health education. Low-quality poisoning-related videos may misinform viewers, potentially delaying appropriate medical care [1]. Healthcare professionals should actively contribute to producing reliable digital content to counteract misinformation. At the policy level, digital platforms share ethical responsibility in ensuring the visibility of evidence-based medical information. Regulatory frameworks and verification systems could help improve the quality and prioritization of educational health videos [14].

This study has several strengths, including its systematic design, the use of multiple validated scoring tools, and the broad inclusion of various poisoning types, which enhances the comprehensiveness of the analysis. However, some limitations should be acknowledged. First, restricting the search to English-language videos may limit the generalizability of the findings to non-English-speaking audiences. Similarly, excluding videos longer than one hour may have resulted in the omission of in-depth educational or lecture-based content. The cross-sectional design provides only a temporal snapshot, preventing assessment of how video quality or popularity evolves over time. In addition, the study did not perform multivariate regression analyses, which could help identify independent predictors of video quality. Future research with multilingual datasets, inclusion of longer format content, and longitudinal or multivariate analytical approaches may yield more comprehensive insights [12,28].

Beyond identifying deficits in video quality, practical solutions are essential for improving digital health communication. Participatory design (co-design) methods have recently emerged as an effective strategy for developing user-centered, credible, and accessible health information resources [30,31]. Engaging healthcare professionals, educators, designers, and target audience members in the creation process may improve clarity, trustworthiness, and uptake of poisoning-related educational videos. Incorporating co-design principles into digital content development could contribute to more sustainable and clinically aligned public health communication.

From an end-user perspective, identifying trustworthy medical content on YouTube remains challenging [10,28]. To assist viewers, several practical criteria can be recommended: prioritizing videos produced or presented by licensed healthcare professionals, verifying the presence of explicit source citations and references, ensuring consistency with established medical guidelines, and favoring content endorsed by reputable institutions. High-quality videos typically demonstrate structured explanations, transparent authorship, and evidence-based recommendations features that viewers should actively seek when selecting educational material.

Beyond individual behavior, platform-level interventions are essential to mitigate the proliferation of low-quality medical videos [14,28]. Digital platforms could implement quality verification indicators, introduce expert-reviewed content labels, and adjust recommendation algorithms to prioritize authoritative medical sources. Establishing formal partnerships with academic institutions and public health authorities may further strengthen the reliability and accessibility of verified content, contributing to safer dissemination of medical information.

## 5. Conclusions

In our study, videos produced or presented by physicians demonstrated significantly higher educational and informational value compared with non-professional sources, yet viewer engagement (VPI) did not correlate with scientific accuracy. Notably, approximately 40% of the evaluated videos fell below minimum quality standards when considering both low- and moderate-quality categories, underscoring a substantial risk of misinformation in poisoning-related content. These findings highlight the need to promote verified, evidence-based medical information on digital platforms. Active involvement of healthcare professionals and academic institutions, coupled with platform-level quality verification systems and oversight mechanisms, will play a critical role in improving the accuracy, visibility, and public accessibility of trustworthy medical content, ultimately helping to mitigate the growing threat of digital health misinformation.

## Figures and Tables

**Table 1:** Video characteristics and quality scores (N = 279).

Video Type, N (%)	
Scorpion Envenomation	51 (18.3)
Insect Bite	64 (22.9)
Mushroom Poisoning	54 (19.4)
Snake Envenomation	53 (19)
General Approach to Poisoning	57 (20.4)
Number of views, Mean ± sd	610,701 ± 4,086,360
Time since upload date (days), Mean ± sd	1838 ± 1237
View ratio (views/day), Mean ± sd	413 ± 3428
Duration (seconds), Mean ± sd	10.53 ± 12.06
Number of likes, Mean ± sd	4321 ± 25,268
Number of comments, Mean ± sd	420 ± 4772
Content Score, Mean ± sd	8.49 ± 3.46
VPI, Mean ± sd	413.23 ± 3428.07
GQS, Mean ± sd	3.53 ± 1.09
GQS group, N (%)	
Low quality (1–2)	55 (19.7)
Moderate quality (3)	58 (20.8)
High quality (4–5)	166 (59.5)
mDISCERN, Mean ± sd	3.53 ± 1.08
JAMA, Mean ± sd	2.63 ± 0.96

GQS: Global Quality Score; JAMA: Journal of the American Medical Association; VPI: Video Power İndex; mDISCERN: modified DISCERN.

**Table 2:** Evaluation in terms of source and category distribution.

Category	N (%)
Educational	212 (76)
Entertainment	20 (7.2)
News and Politics	34 (12.2)
Interviews and Blogs	12 (4.3)
Others	1 (0.4)
Source	
Government/News Agencies	37 (13.3)
Universities/Professional Organizations	30 (10.8)
Physicians and Physician Groups	118 (42.3)
Independent Health Information Websites	39 (14)
Medical Advertisement/Commercial Companies	18 (6.5)
Individual	34 (12.2)
Others	3 (1.1)
Presenter	
Patients	4 (1.4)
Patient’s Family Member/Caregiver	5 (1.8)
Doctor	150 (53.8)
Nurses	25 (9)
Reporter	57 (20.4)
Social Individual/Influencer	31 (11.1)
Others	7 (2.5)

**Table 3:** Comparison of Quality Indices by Poisoning Type.

	Scorpıon (N = 51)	Insect (N = 64)	Mushroom (N = 54)	Snake (N = 53)	General (N = 57)	*p* Value
GQS	3.33 ± 1.01	3.15 ± 1.08	3.03 ± 1.16	3.92 ± 0.95	4.24 ± 0.68	<0.001
JAMA	2.43 ± 0.92	2.43 ± 0.87	2.12 ± 1.02	2.94 ± 0.90	3.24 ± 0.68	<0.001
mDISCERN	3.33 ± 1.01	3.14 ± 1.05	3.01 ± 1.16	3.92 ± 0.95	4.24 ± 0.68	<0.001
VPI	1111.48 ± 7342.38	177.09 ± 1005.47	369.51 ± 1198.01	473.17 ± 2751.37	39.36 ± 154.99	0.060

GQS: Global Quality Score; JAMA: Journal of the American Medical Association; VPI: Video Power İndex; mDISCERN: modified DISCERN.

**Table 4:** Comparison by Educational Status, Source, and Speaker.

Category	Educational (n: 212)	Non-Educational (n: 67)	*p*
mDISCERN	3.89 ± 0.88	2.38 ± 0.88	<0.001
JAMA	2.96 ± 0.79	1.61 ± 0.74	<0.001
GQS	3.90 ± 0.87	2.37 ± 0.90	<0.001
VPI	317.06 ± 3636.05	717.54 ± 2666.27	0.196
Kaynak	Physician (n: 118)	Non-physician (n: 161)	
mDİSCERN	4.15 ± 0.86	3.07 ± 1.01	<0.001
JAMA	3.21 ± 0.76	2.21 ± 0.88	<0.001
GQS	4.16 ± 0.87	3.07 ± 1.01	<0.001
VPI	34.88 ± 89.80	690.53 ± 4497.75	0.92
Speaker	Physician (n: 150)	Non-physician (n: 129)	
mDİSCERN	4.06 ± 0.79	2.90 ± 1.05	<0.001
JAMA	3.12 ± 0.73	2.08 ± 0.91	<0.001
GQS	4.07 ± 0.80	2.90 ± 1.06	<0.001
VPI	26.30 ± 75.49	2863.14 ± 5013.70	0.176

GQS: Global Quality Score; JAMA: Journal of the American Medical Association; VPI: Video Power İndex; mDISCERN: modified DISCERN.

## Data Availability

Data supporting the findings of this study are available from the corresponding author upon reasonable request. The data cannot be shared publicly due to restrictions imposed by YouTube’s terms of service and privacy considerations regarding video ownership.

## Abbreviations

GQS	Global Quality Score
JAMA	Journal of the American Medical Association
mDISCERN	Modified DISCERN
VPI	Video Power Index
SPSS	Statistical Package for the Social Sciences
ED	Emergency Department
